# Supplementary figures and images for: The gut lactic acid bacteria metabolite, 10-oxo-cis-6,trans-11-octadecadienoic acid, suppresses inflammatory bowel disease in mice by modulating the NRF2 pathway and GPCR-signaling
Source: Front Immunol. 2024 Apr 30;15:1374425. doi: 10.3389/fimmu.2024.1374425 (PMC11091332; doi:10.3389/fimmu.2024.1374425)

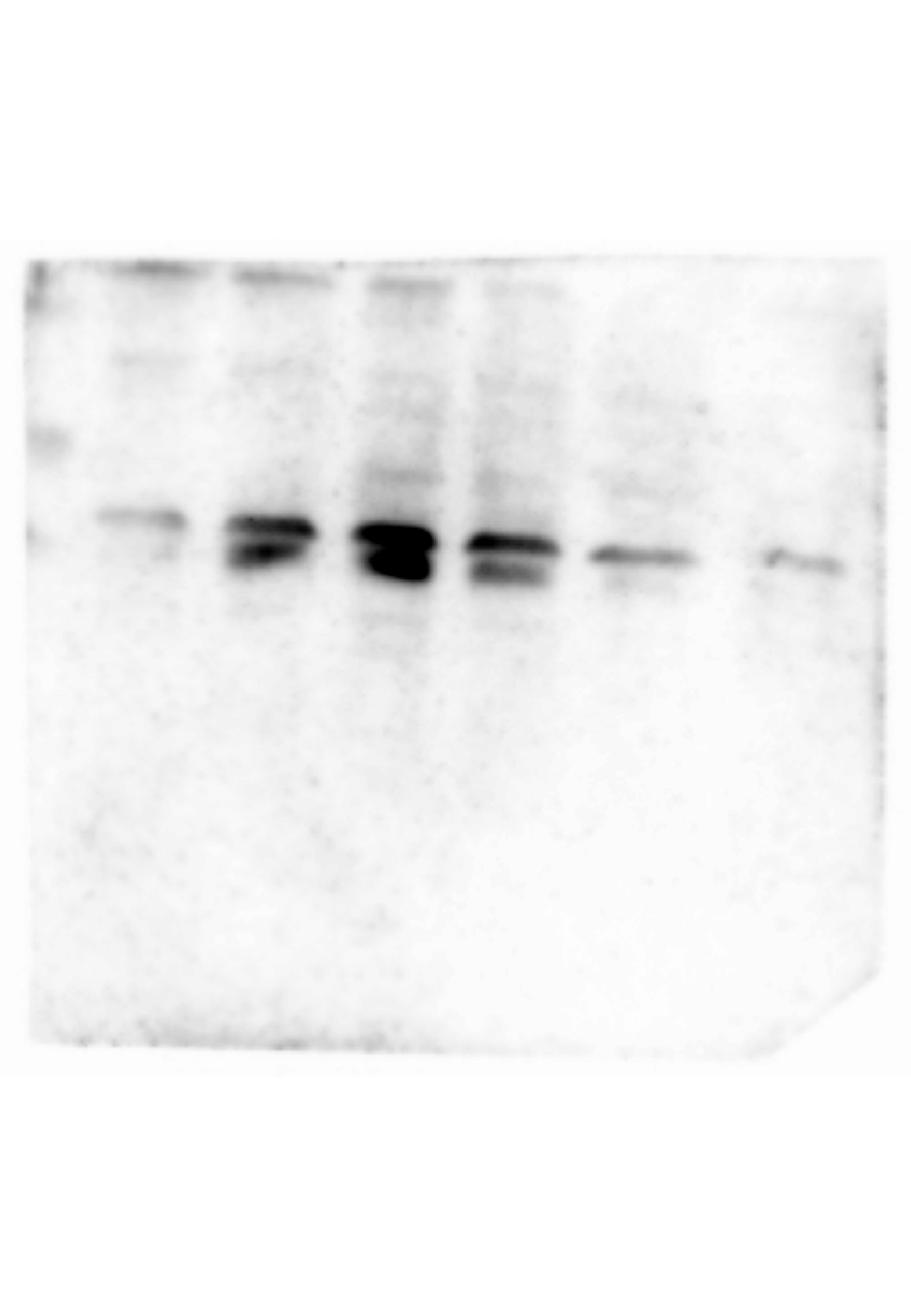

Supplement: Supplementary file 7 [file Image_1.jpeg]

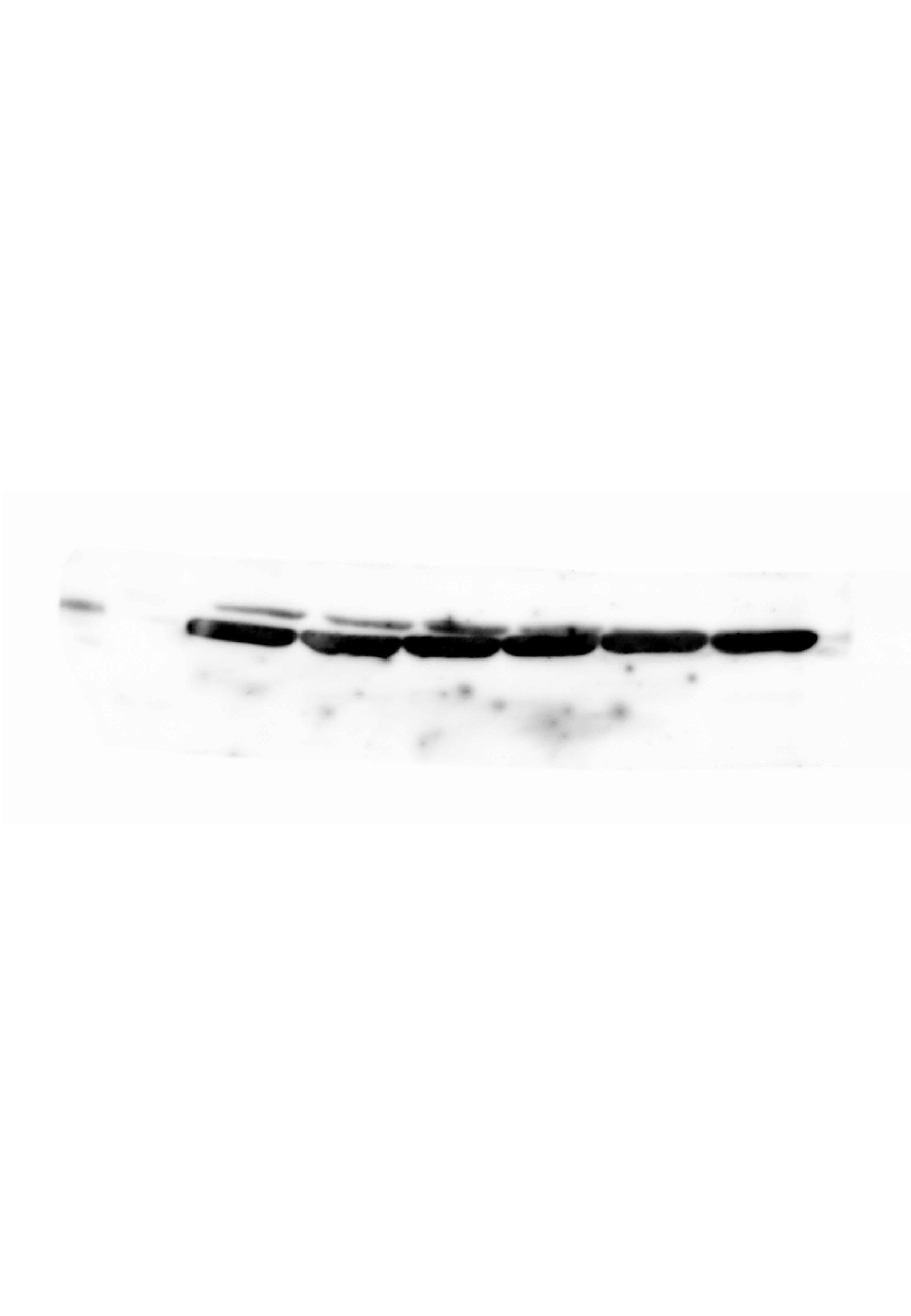

Supplement: Supplementary file 8 [file Image_2.jpeg]
