# Supplementary material for: The gut lactic acid bacteria metabolite, 10-oxo-cis-6,trans-11-octadecadienoic acid, suppresses inflammatory bowel disease in mice by modulating the NRF2 pathway and GPCR-signaling
Source: Front Immunol. 2024 Apr 30;15:1374425. doi: 10.3389/fimmu.2024.1374425 (PMC11091332; doi:10.3389/fimmu.2024.1374425)
Supplement: Supplementary file 9 [file Presentation_1.pdf]

Supplementary Material

Supplementary Figures and Figure Legends.

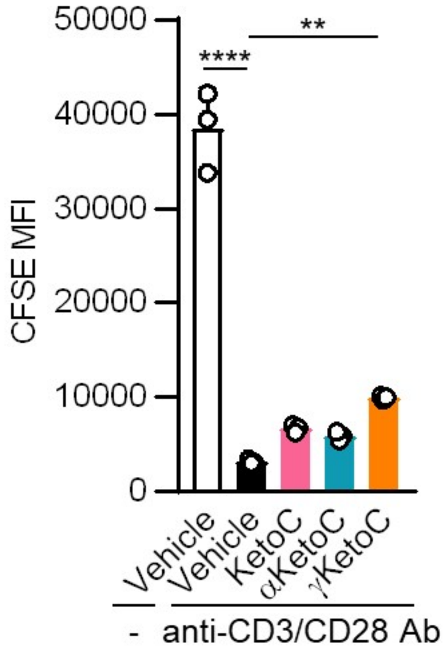

Figure S1. The proliferation of CD4$^+$ T cells stimulated with anti-CD3 and anti-CD28 Abs.

A result in an independent experiment performed under the same condition as that shown in Fig. 1C. Data represent the mean ± SD of triplicate samples. Statistical analysis was performed by the Dunnett's test. ***p* < 0.01, ****p* < 0.005.

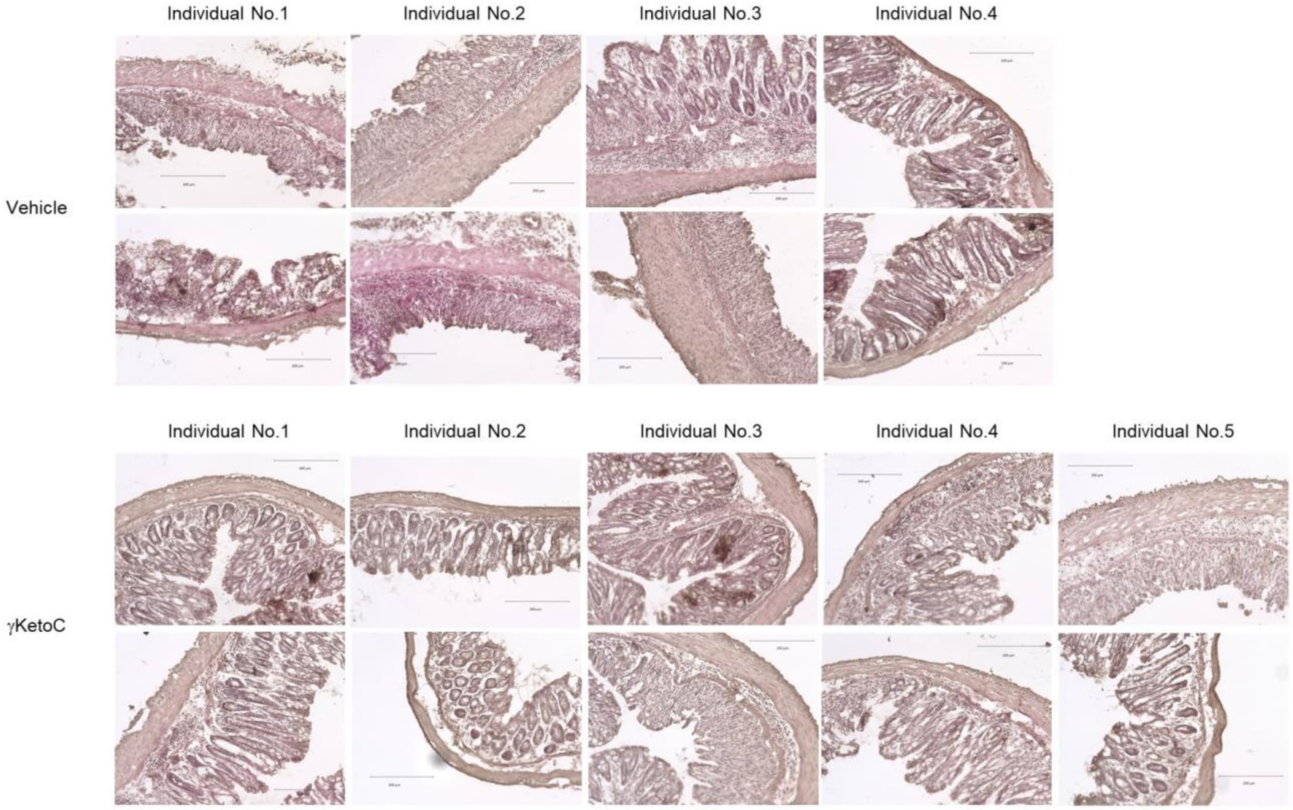

Figure S2. Histology of the colon of colitis induced mice.

Vehicle; DSS treatment without γKetoC administration (n=4), γKetoC; γKetoC administration with the DSS treatment (n=5).
